# Supplementary figures and images for: Dengue and chikungunya virus loads in the mosquito Aedes aegypti are determined by distinct genetic architectures
Source: PLoS Pathog. 2023 Apr 12;19(4):e1011307. doi: 10.1371/journal.ppat.1011307 (PMC10124881; doi:10.1371/journal.ppat.1011307)

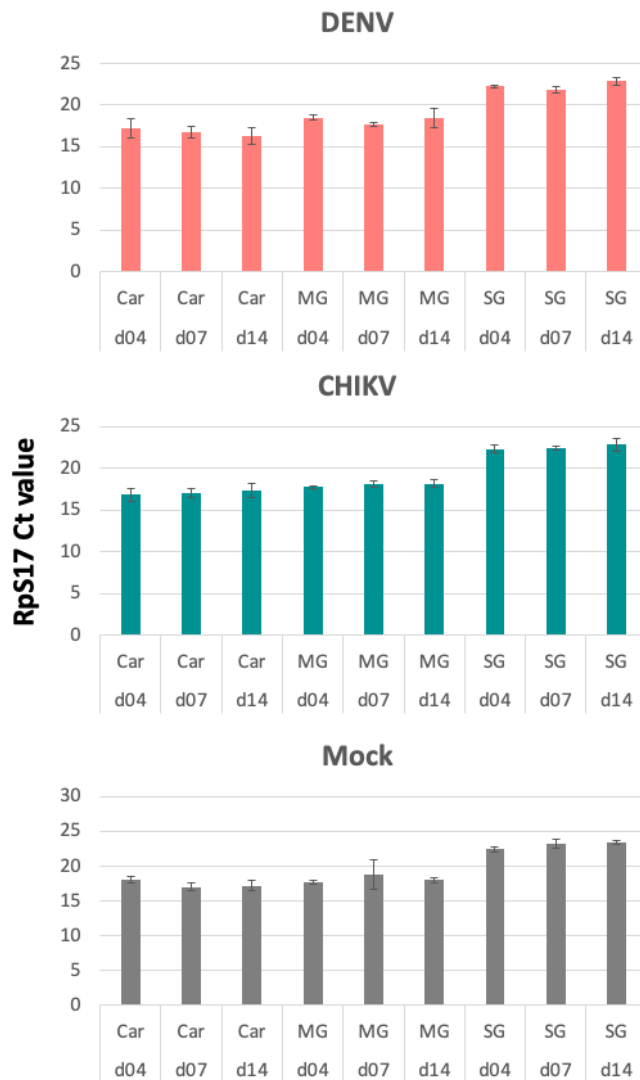

Supplement: S1 Fig — Three tissues, midgut (MG), carcass (Car), and salivary glands (SG), were dissected on three different days post-infection (dpi). (PDF) [file ppat.1011307.s001.pdf]
