# Supplementary material for: Dengue and chikungunya virus loads in the mosquito Aedes aegypti are determined by distinct genetic architectures
Source: PLoS Pathog. 2023 Apr 12;19(4):e1011307. doi: 10.1371/journal.ppat.1011307 (PMC10124881; doi:10.1371/journal.ppat.1011307)
Supplement: Attachment — Submitted filename: Novelo et al Response to ReviewersFeb1.pdf [file ppat.1011307.s002.pdf]

Dear editor,

Thank you for mediating the differences in reviews and highlighting a list of changes we should incorporate. We have addressed the list of concerns and it has improved the manuscript.

See below as highlighted by the editor:

Please make your RNA-seq datasets publicly available by depositing your datasets in a publicly accessible repository
Response. Done. Uploaded to figshare DOI (listed in PP online system) and published. 10.6084/m9.figshare.20483691

Please add a table or a graph with the Ct values for RpS17 and its standard curve to assess the consistency of this housekeeping gene among tissues and mosquitoes for the normalization of the qPCR data.
Response. We provided a graph of Ct values for RpS17 by treatment in the supporting materials showing they do not vary across treatments (infected vs mock) for any of the time points, and tissues. As this is relative quantification, there is no standard curve.

Specify how many mosquitoes were used for the tissue-specific expression of AEL004181 experiment and from what mosquito families.
Response. Information now added in multiple places – figure legend, methods, and results

Make sure to include the Table 1 (primers and probes), which is currently missing.
Response. Done.

Please explain what “High and Low CHKV families” are in the introduction (even if it is explained in the Results section).
Response. Done.

Clarify the description of the gene LOC110676965 in the results section. It is first described as encoding a Zinc finger containing protein (line243) but is later described as a long non coding RNA (lncRNA, line258).
Response. Done. Non-coding RNA was in error.

The following is a more minor point:
Consider adding a schematic diagram to summarize the main experimental design with the breeding approach and infection experiments.
Response. Done. New fig 1 added.

Additional small corrections

Line 68: Should be ‘Arthropod-borne’
Response. Done

Ln. 361. Speaking of MSVs, did the authors (or anyone) screen these populations of Ae. aegypti from Monterrey for ISVs? The presence of MSFs that are flaviviruses or alphaviruses could have had relevance for the observations in the present study.
Response. No, we did not.

Ln. 419. You could clarify here that you mean individualized gene editing for controlling different pathogens would be needed.
Response. Changed individualized to ‘virus-specific’

Ln 429. I don’t understand how an F3 line of Ae. aegypti collected in Monterrey, Mexico was then reared for another three generations to adapt to colony feeding. Does this mean someone in Monterrey started this colony in Mexico for three generations, and then the F3s were shipped to Penn State? It would help to clarify this history. Is there a reference for the collection of *Ae. aegypti* eggs from traps (I assume these are ovitraps) in Monterrey that can be cited?
Response. Better described. No, there is no reference for a field collection.

Ln. 480. If specifying where CHIKV challenges were conducted, would be good to clarify where the DENV challenged occurred.
Response. Done

Ln. 555. Would be good to clarify that the Trizol was part of the RNA extraction step as described above.
Response. Done

Figure 1, explains in detail in the experiments why only 37 families were taken if they could have been more considering that I have understood that 600 mosquitoes were placed individually, and according to details of the methods have been used only those that laid more than 60 eggs. Is that why only 37 have remained?
Response. Done. Improved with the addition of an experimental design figure and an expanded explanation in what is now figure 2 legend.
